# Homogentisate 1-2-Dioxygenase Downregulation in the Chronic Persistence of *Pseudomonas aeruginosa* Australian Epidemic Strain-1 in the CF Lung

**DOI:** 10.1371/journal.pone.0134229

**Published:** 2015-08-07

**Authors:** Christopher J. Harmer, Matthew Wynn, Rachel Pinto, Stuart Cordwell, Barbara R. Rose, Colin Harbour, James A. Triccas, Jim Manos

**Affiliations:** 1 Bacterial Pathogens in Cystic Fibrosis Group, Department of Infectious Diseases and Immunology, Central Clinical School, The University of Sydney, Sydney, Australia; 2 School of Molecular Biosciences, The University of Sydney, Sydney, Australia; 3 Microbial Pathogenesis and Immunity Group, Department of Infectious Diseases and Immunology, Central Clinical School, The University of Sydney, Sydney, Australia; University of North Dakota, UNITED STATES

## Abstract

Some *Pseudomonas aeruginosa* strains including Australian Epidemic Strain-1 (AES-1 or AUS-01) cause persistent chronic infection in cystic fibrosis (CF) patients, with greater morbidity and mortality. Factors conferring persistence are largely unknown. Previously we analysed the transcriptomes of AES-1 grown in Luria broth, nematode growth medium for *Caenorhabditis elegan*s assay (both aerobic) and artificial sputum medium (mainly hypoxic). Transcriptional comparisons included chronic AES-1 strains against PAO1 and acute AES-1 (AES-1R) against its chronic isogen (AES-1M), isolated 10.5 years apart from a CF patient and not eradicated in the meantime. Prominent amongst genes downregulated in AES-1M in all comparisons was homogentisate-1-2-dioxygenase (*hmgA*); an oxygen-dependent gene known to be mutationally deactivated in many chronic infection strains of *P*. *aeruginosa*. To investigate if *hmgA* downregulation and deactivation gave similar virulence persistence profiles, a *hmgA* mutant made in UCBPP-PA14 utilising RedS-recombinase and AES-1M were assessed in the *C*. *elegan*s virulence assay, and the C57BL/6 mouse for pulmonary colonisation and TNF-α response. In *C*. *elegans*, *hmgA* deactivation resulted in significantly increased PA14 virulence while *hmgA* downregulation reduced AES-1M virulence. AES-1M was significantly more persistent in mouse lung and showed a significant increase in TNF-α (p<0.0001), sustained even with no detectable bacteria. PA14Δ*hmgA* did not show increased TNF-α. This study suggests that *hmgA* may have a role in *P*. *aeruginosa* persistence in chronic infection and the results provide a starting point for clarifying the role of *hmgA* in chronic AES-1.

## Introduction

Persistent *P*. *aeruginosa* infections are the leading cause of morbidity and mortality in cystic fibrosis patients. Patients are typically transiently infected with different strains of *P*. *aeruginosa* throughout infancy and adolescence, before the establishment of chronic infection in adolescence. Whilst characteristics such as a hypermutator phenotype [1] exotoxin production [2] and regulation of genes involved in energy metabolism and biofilm formation [3, 4] have been shown to confer a selective advantage to *P*. *aeruginosa* in the CF lung, the precise mechanisms permitting long term persistence in what is normally a sterile environment are largely unknown.

The Australian Epidemic Strain-1 (AES-1 or AUS-01) is recognised as a hypervirulent persistent transmissible frequent clonal strain (FCC) that is endemic in CF clinics on the Eastern Australian seaboard [5, 6]. Several studies by our group have investigated AES-1 isolates and compared these with *P*. *aeruginosa* PAO1, non-epidemic strains (infrequent clones or IFCC) [7, 8]. We also compared acute and chronic isogens (AES-1R and AES-1M, respectively) from the same patient taken 10.5 years apart and not eradicated in the interim [9]. One of the few genes that demonstrated consistent downregulation in chronic AES-1 in all studies was homogentisate-1-2-dioxygenase (*hmgA* AES1R02079-AES-1R sequence: GenBank Accession number AFNF00000000). We first compared the transcriptomes of four chronic AES-1 isolates from different CF patients against PAO1 grown aerobically in Luria broth and found a statistically significant downregulation of *hmgA* in all planktonic AES-1 compared to planktonic PAO1 (-4.9×, B-statistic = 1.05) (Table 1). At the same time, three out of four non-AES-1 chronic isolates micro-arrayed showed an increased expression of *hmgA* compared to PAO1 [8]. Additionally, isogens of an AES-1 isolate (C7) collected at early and chronic infection and grown aerobically on nematode growth medium for *Caenorhabditis elegan*s assay and microarrayed in duplicate showed a downregulation (-2.3×; Table 1) of *hmgA* in the chronic isogen, though p>0.05 [7]. *hmgA* was also prominent amongst genes downregulated between acute (AES-1R) and chronic (AES-1M) isogens of AES-1 from the same patient taken 10.5 years apart and not eradicated in the interim (-7.2×, p = 0.03) (Table 1) [9]. In this case, genes were identified using a non-redundant array (PANarray) and growth in a medium that closely mimics cystic fibrosis lung sputum (ASMDM) [10].

**Table 1:** Differential expression of *hmgA* by *P*. *aeruginosa* AES-1 by microarray analyses (all experiments conducted in biological duplicate).

Microarray Comparison	Growth conditions	Fold-change	Publication
Chronic AES-1M vs acute AES-1R in **ASMDM**	Anaerobic/micro-aerophilic	-7.2×*	[9]
Chronic AES-1M vs acute AES-1R in-**L-broth**	Aerobic	-3.3×	Unpublished data
Chronic AES-1 vs PAO1 in **L-broth**	Aerobic	-4.9×*	[8]
Chronic AES-1 vs non-clonal strain on **NGM medium**	Aerobic	-2.3×	[7]

*Statistically significant.

Thus our data indicate that *hmgA* downregulation is a feature of both aerobic and anaerobic AES-1 growth, despite evidence that *hmgA* is oxygen-dependent and together with 4-hydroxyphenylpyvruvate dioxygenase (*hpd*) is possibly indirectly repressed by the anaerobic nitrate regulator (ANR) during anaerobic growth [11–13]. HmgA and Hpd are also involved in the degradation of the aromatic amino acids L-phenylalanine and L-tyrosine, which serve as a carbon source for *P*. *aeruginosa* in CF sputum [14]. The transcriptional regulator PhhR is known to regulate expression of *hmgA*, *hpd* and several other aromatic amino acid catabolism genes in *Pseudomonas putida* [15], and a similar role has been proposed in *P*. *aeruginosa* [16].

This downregulation, but not mutational inactivation, as found in other chronic strains [17] (all fluorescence values were above the ‘absent’ cut-off for expression) of *hmgA* in chronic AES-1 strains (Table 1) may represent an important new indicator of the mechanisms of persistence of epidemic strains of *P*. *aeruginosa* such as AES-1. There was no evidence of pyomelanin hyperproduction as a result of *hmgA* downregulation in any AES-1 strain examined on agar, suggesting that while pyomelanin does play a role in virulence [18] it does not play a role in adaptation/persistence, since AES-1M showed increased in vivo persistence. In order to better understand *hmgA*, we have looked at the in vitro and in vivo effects of *hmgA* downregulation in AES-1 and also at *hmgA* inactivation by mutagenesis. The mutagenesis method utilised in construction of the Δ*hmgA* was pioneered by Lesic et al [19], and couples a previously published but rarely used method (lambda Red recombinase) to improve the speed and efficiency of mutant generation, with a well established gene replacement method of generating mutants in *P*. *aeruginosa* isolates [20].

## Materials and Methods

### 2.1 Bacterial strains and plasmids

AES-1R was isolated from a child aged 14 months at the same time as the deaths of five CF-infants infected with AES-1 [21]. AES-1M was isolated from the same patient at age 11 years 9 months. The AES-1 was not eradicated in the patient in the intervening period. Both isogens were kindly provided by D. Armstrong, Royal Children’s Hospital, Melbourne, Victoria, Australia. Written informed consent was obtained from the patient's next of kin by the Royal Children’s Hospital and The Southern Health Service, Melbourne, Victoria, Australia. Ethics approval for this study was given by the University of Sydney Human Research Ethics Committee (Protocol Number: X07-0029, Reference Number 6999).

Mutagenesis was carried out in a UCBPP-PA14 (PA14) background due to its favourable antibiotic susceptibility profile and efficient transformation frequency compared to AES-1. PA14 is a widely studied burns isolate [22] and previous studies have used the PA14 strain to establish growth criteria in ASMDM and CF sputum [10, 23, 24]. Furthermore, PA14-like clones have been isolated from CF patients in Europe [25].

The lambda Red recombinase-containing plasmid pUCP18-RedS was utilised to drive homologous recombination and improve its efficiency [19]. The *Escherichia-Pseudomonas* shuttle vector pUCP20 (kindly provided by H. Schweizer, Colorado State University, USA) was used to generate complementation constructs. pPS856 (H. Schweizer) contained a gentamicin resistance cassette used to facilitate mutant selection in *P*. *aeruginosa*. The gentamicin cassette is flanked by Flippase Recombination Target (FRT) sites, allowing downstream generation of unmarked mutants through the use of flippase (*flp*) to excise the cassette following mutagenesis.

### 2.2 Generation of mutants via three-way PCR

PCR procedures were performed using *Pfu* proofreading polymerase (Life Technologies) according to manufacturer’s instructions. Primer pairs (Table 2) were used to amplify ca. 500 bp regions upstream and downstream of the target gene and thus create external ‘arms’ for homologous recombination. All primers were designed based on the PA14 reference sequence (GenBank accession number CP000438) after first confirming that the sequence of the *hmgA* gene in AES-1R was identical to that in PA14. The forward primer for the upstream region and the reverse primer for the downstream region were normal 18–20 bp oligos. In addition to the 18–20 bp sequence required to amplify the region of interest, the reverse primer for the upstream region and the forward primer for the downstream region also incorporated 20–25 bp overlaps homologous to the 5’ and 3’ ends of the gentamicin cassette in pPS856 respectively to allow the arms to bind to the cassette in the three-way overlap-extension PCR amplification.

**Table 2:** Oligonucleotide primers used in this study.

Primer name	Sequence (5’-3’)
**Mutagenesis primers**	
pPS856-GmF^a^	CGAATTAGCTTCAAAAGCGCTCTGA
pPS856-GmR^a^	CGAATTGGGGATCTTGAAGTTCCT
*hmgA*-UpF^b^	AAGCAGGTCTCGTTGAGCAC
*hmgA*-UpR-Gm^c^	TCAGAGCGCTTTTGAAGCTAATTCGTGGCTGCGTTATTTTTATCG
*hmgA*-DnF-Gm^d^	AGGAACTTCAAGATCCCCAATTCGGACTTTCCCCTGCAAAACCT
*hmgA*-DnR^e^	ATGGGCACGTGCTTGTAGTT
**Complementation primers**	
*hmgA*-compF	GGAGGTGAGCTCGGGACTGCTGTTCTTTTCCA
*hmgA*-compR	ATGCCCGGATCCAGGTTTTGCAGGGGAAAGTC
**qPCR primers**	
*nadC*-forward	CGGCGGCGACCTTACC
*nadC*-reverse	CCTGGCGGAAGACCTCGTC
*hmgA*-forward	CTGTACGCCGAACTGCTCTC
*hmgA*-reverse	GTGATGCGGTATAGCCAGGT

^a^ Sourced from Choi and Schweizer, 2005.

^b^ *gene*-UpF: Regular 18–20bp forward primer upstream of the target gene

^c^ *gene*-UpR-Gm: 18–20bp reverse primer upstream of the target gene incorporating a 20–25bp overlap homologous to the 5’ end of the gentamicin resistance cassette in pPS856

^d^ *gene*-DnF-Gm: 18–20bp forward primer downstream of the target gene incorporating a 20–25bp overlap homologous to the 3’ end of the gentamicin resistance cassette in pPS856

^e^ *gene*-DnR: Regular 18–20bp forward primer downstream of the target gene.

The three-way overlap-extension PCR contained as templates the two arms and the gentamicin resistance cassette isolated from pPS856, and generated a continuous PCR product containing the cassette flanked by the 500bp arms. After sequencing to determine correct orientation and fidelity of sequence, the entire construct was electroporated into *P*. *aeruginosa* rendered electrocompetent and expressing the lambda RedS recombinase from pUCP18-RedS. Replacement of the target gene with the construct containing the gentamicin resistance cassette via homologous recombination was detected after overnight growth at 37°C on nutrient agar supplemented with gentamicin (50μg/ml). Mutant strains were cured of the pUCP18-RedS plasmid by counter-selection on nutrient agar supplemented with 5% (w/v) sucrose. Knockout of the gene of interest was confirmed by sequencing the left and right junctions in each mutant strain.

Complemented strains were generated by first amplifying the wild type and *hmgA* target gene using primers listed in Table 2. The forward primers included a *BamH*I site at their 5’ end and the reverse primers included a *Sac*I site at their 5’ end to facilitate ligation into the pUCP20 complementation vector. Each vector was sequenced and transformed into the mutant strain to restore expression of the target gene.

### 2.3 Phenotypic and genotypic tests

#### 2.3.1 Validation of gene expression by qPCR

The level of *hmgA* expression by qPCR for AES-1R and AES-1M was determined by quantitative SYBR-green-PCR (qPCR) using a Rotor-Gene 6000 Real-Time amplification system (Qiagen-Corbett Life Sciences, Australia) [9]. The same method was used here for *ΔhmgA* strains. Briefly, after overnight growth at 37°C, total RNA was extracted from Δ*hmgA* strains using the Qiagen RNeasy extraction kit according to manufacturers’ instructions (Qiagen, Australia), and reverse transcription using 50U SuperScriptII (Invitrogen, Australia) was carried out. Oligonucleotide primers (Table 2) were used to quantify the expression of *hmgA* relative to the *nadC* endogenous control. All samples were analysed in triplicate, and each analysis was repeated in biological duplicate.

#### 2.3.2 Caenorhabditis elegans slow-killing virulence assay

The *C*. *elegans* slow-killing virulence model utilized strain CF512 (provided by the Caenorhabditis Genetics Centre, University of Minnesota, USA) and was performed as previously described [7] Experiments for AES-1R and AES-1M were performed with six biological replicates and for PA14 and PA14*ΔhmgA* in biological triplicate, and results presented as the mean of the readings. The log-rank (Mantel-Cox) test was used to analyse survival curves based upon the percentage of survivors at the sampled time points.

#### 2.3.3 Virulence factor assays

A panel of standard phenotypic tests were carried out as previously described [7]. Briefly, AES-1R, AES-1M, PA14 and PA14Δ*hmgA* were tested for pyocyanin, pyoverdine, elastase, protease, rhamnolipid. Experiments were performed in biological triplicate and results presented as the mean of the triplicates.

### 2.4 Mouse model and flow cytometry studies

All mice used in this study were 8–12 week old female C57BL/6 mice sourced from the Animal Resources Centre (Western Australia, Australia). Mice were housed at the University of Sydney Bosch Rodent Facility and maintained under specific pathogen free conditions. All animal experiments were approved by the University of Sydney Animal Ethics Committee (Approval no. K00/1-2010/2/5237).

In preparation for sacrifice, mice (three mice per group per time point) were anaesthetised with 5% (v/v) isofluorane over 1.8L/min oxygen and a bacterial dose of 10^6^ CFU was administered to the nares. At set time points (3-, 24- and 72-hrs post-infection) the animals were sacrificed by cervical dislocation, lungs and spleen were removed processed using standard protocols for bacterial counts and flow cytometry analysis. Animals were monitored for the whole experimental time-course and clinical sign scores were recorded on a daily basis. Three signs were monitored: hunched posture, starry coat, and lethargy. Each sign was monitored in accordance with the following scale: “0” denoted no signs; “1,” initial signs; and “2,” fully exhibited signs. The individual scores for the three signs were combined to give a maximum score of six. If any mice exhibited the maximum clinical score they were immediately euthanased.

Single cell suspensions of mouse lung homogenates were stained in 50μl FACSwash containing pre-titered monoclonal antibodies (CD45.2, B220, Ly6G, CD11b, CD4, CD8, (BD Pharminigen) and fixed with 10% (v/v) buffered formalin. Flow cytometry data were acquired on a FACSCanto-II (BD, USA) equipped with violet (405nm), red (633nm) and blue (488nm) lasers. Greater than two million events were acquired for each sample. Data were collected in uncompensated digital format. For analysis of digital data, a software compensation matrix was calculated in FlowJo (TreeStar Inc, USA) using single colour controls and applied to each sample. A general gating strategy was employed for the analysis of flow cytometry data. An FSC-Area versus FSC-Height gate, followed by a side scatter-Area versus side scatter-Height gate was first used to exclude any cell doublets from further analysis. This was followed by a wide size gate (SSC-A vs FSC-A) to gate on lymphocytes. After selection of an appropriate lymphocyte gate, bone-marrow derived host cells were selected based on the expression of CD45.2. From the CD45.2^+^ population, B cells were identified by their B220^+^ phenotype, neutrophils by their CD11b^+^Ly6G^+^ phenotype, and T cells by their CD4^+^CD8^-^ or CD4^-^CD8^+^ phenotypes.

## Results

### 3.1 Generation of *hmgA* mutant

For *hmgA*, primers *hmgA*-UpF/*hmgA*-UpR-Gm and *hmgA*-DnF-Gm/*hmgA*-DnR (Table 2) amplified a 465 bp region upstream of the *hmgA* gene and a 440 bp region downstream of the gene in PA14, constituting the left and right ‘arms’. The two products were combined with the FRT-Gm cassette to produce a 1909 bp product consisting of the FRT-Gm cassette flanked by the left and right *hmgA* arms. This product was transformed into PA14 expressing the RedS recombinase, and mutants generated via homologous recombination. PCR amplification and sequencing of the region surrounding *hmgA* produced a 2271 bp fragment in PA14 WT, whilst the Δ*hmgA* mutants possess a gene product of 1909 bp as a result of the 1415 bp *hmgA* gene being replaced with the 1053 bp FRT-Gm cassette. PA14Δ*hmgA* mutants demonstrate accumulation of the brown pigment pyomelanin and this was used in phenotypic characterisation of Δ*hmgA* mutants.

PCR amplification and cloning of the wild type *hmgA* gene into the complementation vector pUCP20 [20] generated plasmid pUCP20-*hmgA* (5448 bp). This plasmid was transformed into PA14Δ*hmgA* to generate a complemented strain. The successful reversion of the mutant strain to a wildtype phenotype following complementation confirmed that this is a non-polar mutation. The complemented strain did not show production of pyomelanin, providing evidence that *hmgA* expression had been restored.

### 3.2 Genotypic and phenotypic tests

#### 3.2.1 *hmgA* transcription by qPCR

Analysis of gene expression by qPCR showed that the Δ*hmgA* strain no longer expressed the target gene, as shown by a large-magnitude (~10^4^) reduction in expression relative to PA14 (not shown). The complemented strain displayed expression comparable to that of the WT strain (+1.2-fold). *hmgA* expression was measured in a different set of ASMDM extracts by qPCR, and its expression in AES-1M was also reduced significantly compared to that in AES-1R (-2.8×) [9].

#### 3.2.2 In vitro virulence of AES-1M and PA14Δ*hmgA*

**Fig 1:**
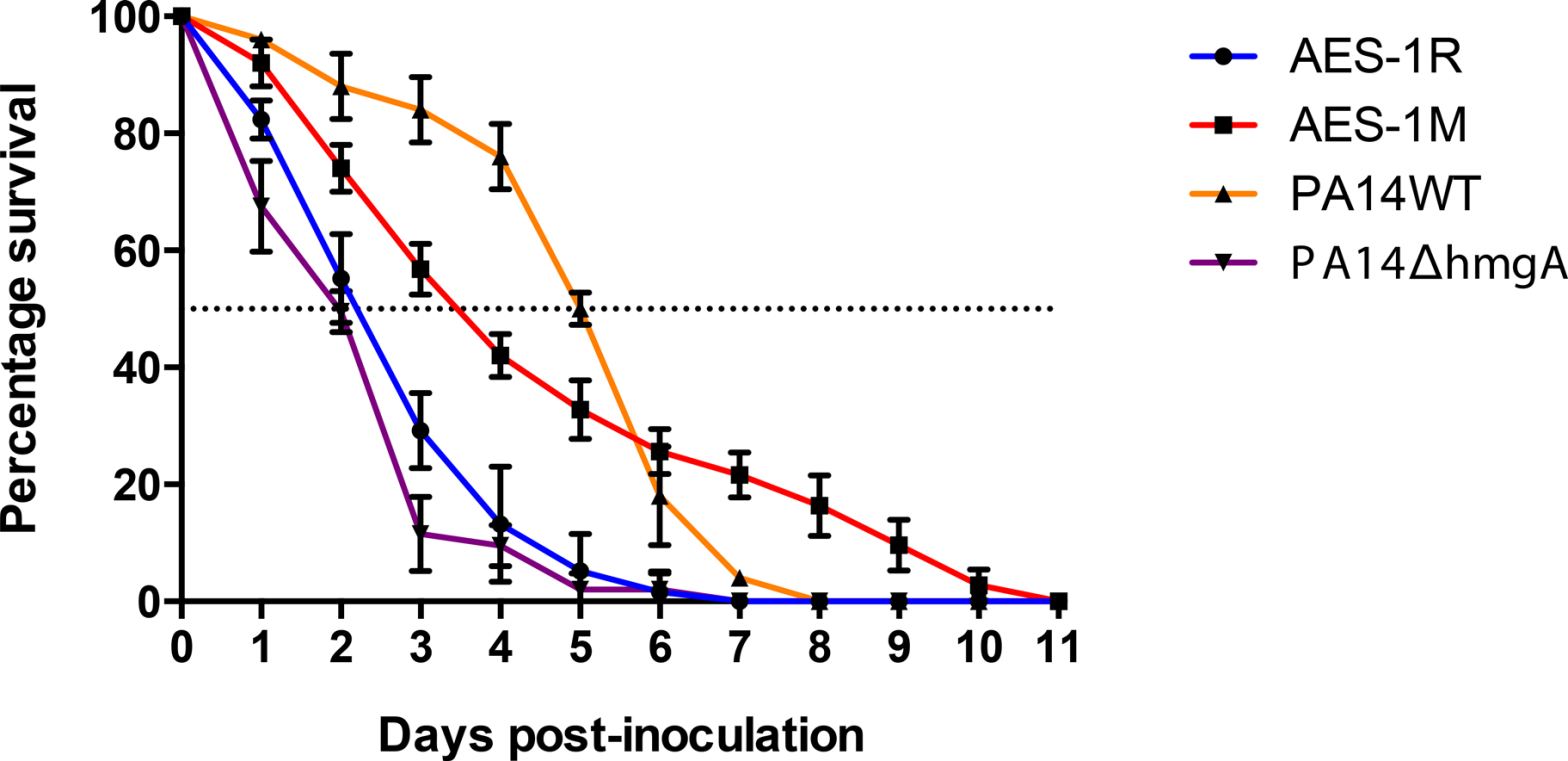
*C*. *elegans* virulence assay. Percent survival of *C*. *elegans* (n = 50) when grown on a lawn of *P*. *aeruginosa* AES-1R, AES-1M, PA14 and PA14Δ*hmgA*. The dotted line indicates the LT_50_ (mean time at which 50% of the worms were killed by ingesting *P*. *aeruginosa*). The individual points are the means of six biological replicates for AES-1 stains and three biological replicates for PA14 strains. The error bars represent the standard error of the means.

In the *C*. *elegans* slow-killing virulence model (Fig 1), the acute isogen AES-1R was more virulent than its chronic counterpart AES-1M, with an LT_50_ 2.1 days against 3.4 days, respectively. While not statistically significant (p = 0.068), this is the opposite of what occurred in the PA14 assay, where the mutant PA14Δ*hmgA* was significantly more virulent than wild type PA14 (p<0.01), with an LT_50_ of 2 days, compared with 5 days for wild type PA14.

Phenotypic assays of AES-1R and AES-1M (not shown) demonstrated a typical chronic isolate pattern of downregulation/deactivation of virulence factors: protease, rhamnolipid, elastase and pyocyanin were not expressed in AES-1M, while pyoverdine remained unchanged. These phenotypic assays were also conducted on PA14 and PA14Δ*hmgA*, and did not identify any significant phenotypic differences (p<0.05) between mutant and wildtype strains.

### 3.3 Mouse model and flow cytometry studies

#### 3.3.1 Persistence of AES-1M and PA14Δ*hmgA* in mouse lung

**Fig 2:**
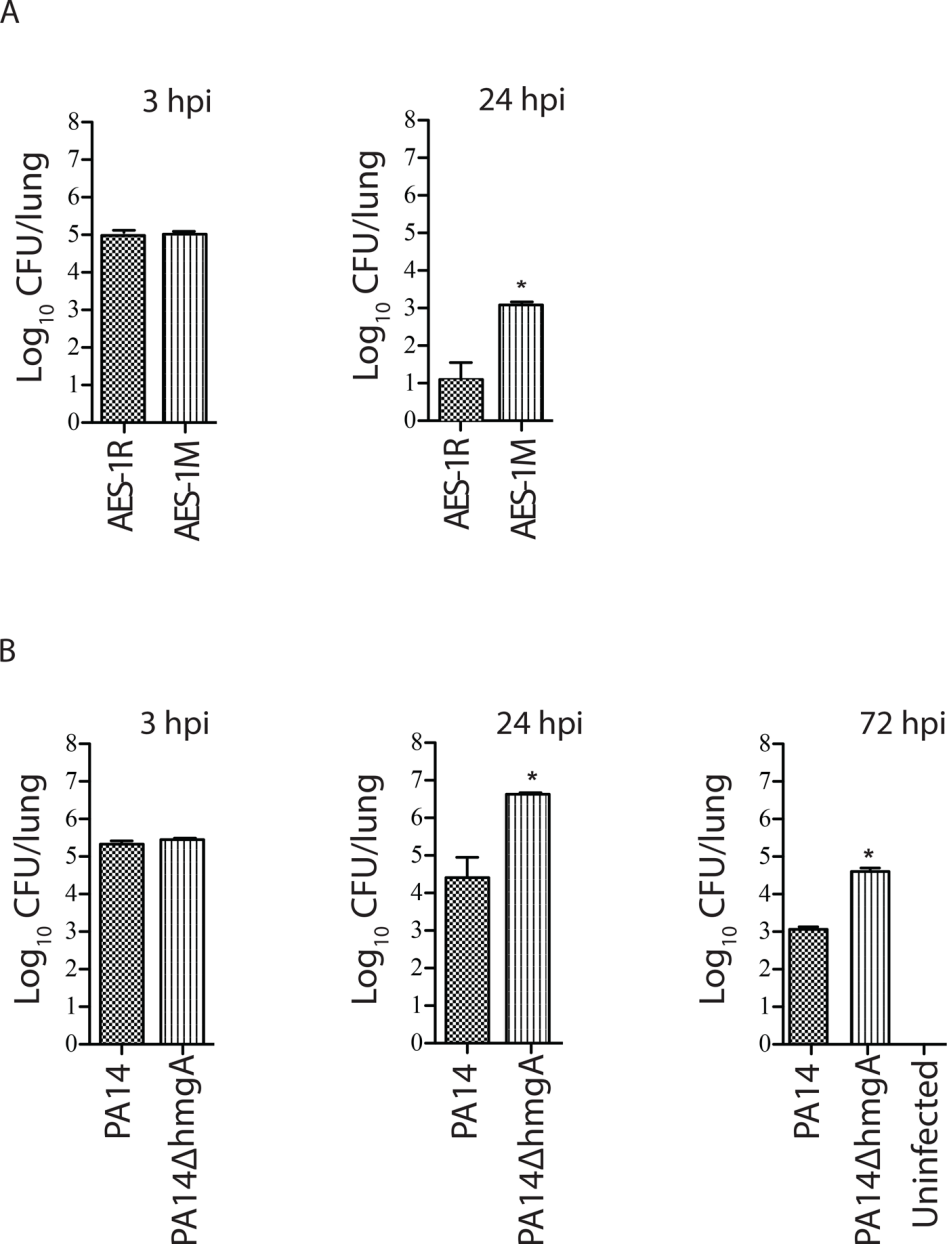
Persistence of *P*. *aeruginosa* AES-1R, AES-1M and PA14, PA14*hmgA*, in a C57BL/6 mouse model. **A**. Mice were infected with 10^6^ CFU of AES-1R and AES-1M and lungs examined at 3-hrs and 24-hrs and 72hrs post-infection for bacterial load (latter not shown). **B.** Mice were infected with 10^6^ CFU wildtype PA14 and PA14*hmgA* and lungs were examined at 3-hrs, 24-hrs, and 72-hrs post-infection for bacterial load. AES-1M was recovered at significantly higher level compared to AES-1R at 24-hr. PA14Δ*hmgA* was recovered at a significantly higher level compared to the wild type PA14 at 24- and 72-hrs post-infection. The significances of differences between strains were determined by ANOVA. 4A: *p<0.001vs AES-1R; 4B* p<0.0001 vs. PA14 WT.

AES-1R and AES-1M showed no significant differences in lung bacterial load at 3-hrs post inoculation indicating that they had similar seeding efficiencies, however by 24-hrs post inoculation, significantly higher (p<0.001) bacterial loads of AES-1M (>3log_10_ CFU/ml) were recovered compared to AES-1R at 1log_10_ CFU/ml (Fig 2A). At 72-hrs post inoculation lung bacterial loads of both AES-1R and AES-1M had declined to <1log_10_ CFU/ml (not shown).

At the three-hr time point there were no significant differences in the lung bacterial load between PA14 wild type and PA14Δ*hmgA*, indicating that the mutation had not impacted upon the colonisation efficiency following intranasal inoculation (Fig 2B). At 24-hrs post inoculation, PA14Δ*hmgA* was present in the lungs at a significantly higher (p<0.0001) load than PA14 wild type. By 72-hrs post-infection, PA14Δ*hmgA* was showing a distinct persistence phenotype compared to PA14 wild type, persisting at a load approximately two logs higher than the wild type (p<0.001). At the 24- and 72-hour time points, mice infected with PA14 wild type all displayed significant signs of clinical illness (mean 3), whilst the PA14Δ*hmgA*-infected mice displayed milder clinical signs (average score 1).

#### 3.3.2 TNF-α response to AES-1M and PA14Δ*hmgA*

**Fig 3:**
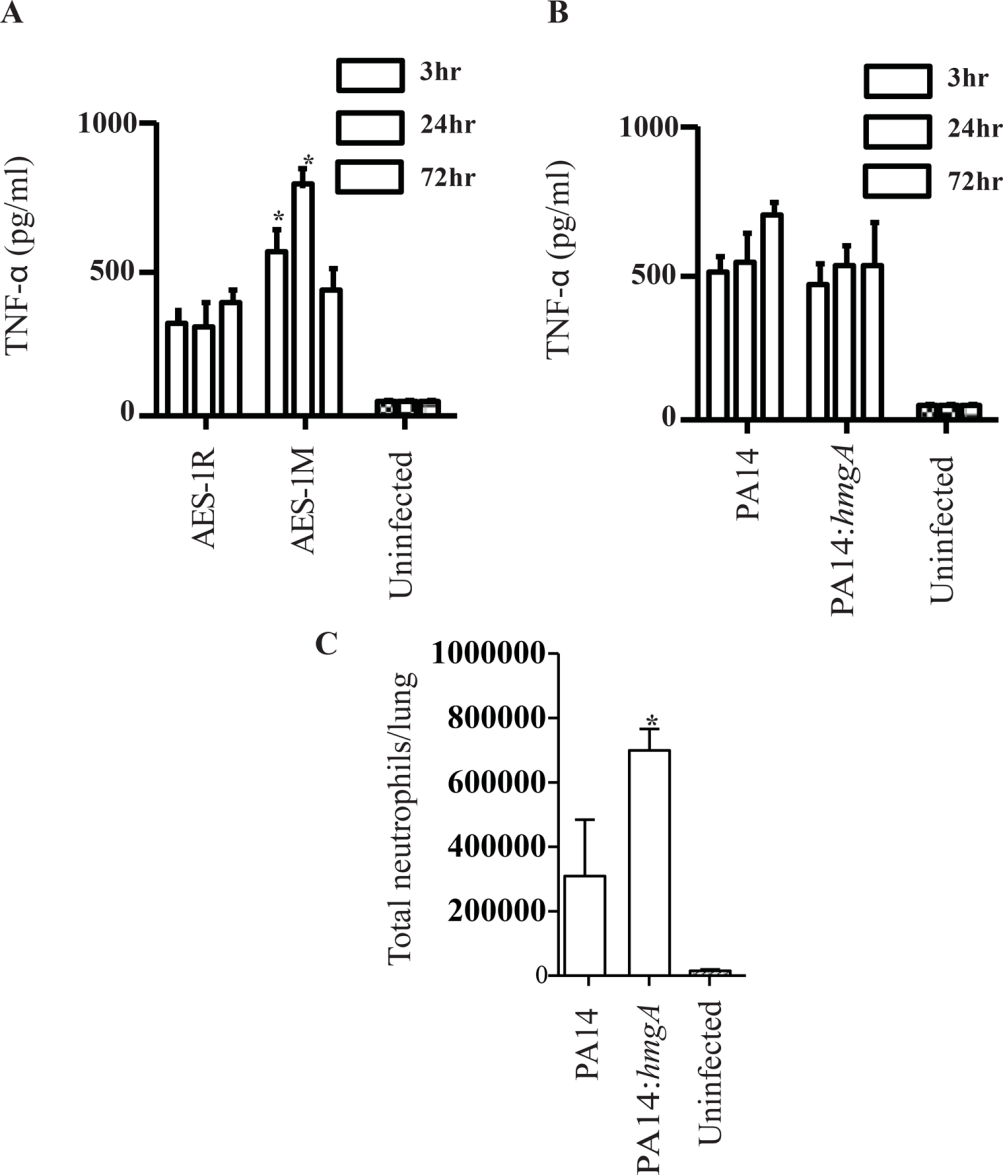
Mouse TNF-α response to lung infection with AES-1R, AES-1M, PA14 and PA14Δ*hmgA*, and neutrophil populations in PA14 and PA14Δ*hmgA*-infected mice. The TNF-α profile of *P*. *aeruginosa*-infected mice was determined in lung homogenates at 3-, 24- and 72-hrs post-infection by ELISA. **A.** AES-1M elicited a rapid and significantly greater TNF-α response compared to AES-1R at 3 and 24-hr, and both responses were still at >400pg/ml at 72hr despite the absence of bacteria in lungs. **B.** Both PA14 and PA14*hmgA* elicited a rapid TNF-α response compared to control uninfected mice by three hrs post-infection however there was no significant difference between them. The significances of differences between strains were determined by ANOVA * p<0.0001 vs. AES-1R. **C.** Mice were infected with 10^6^ CFU of either wildtype PA14 or PA14Δ*hmgA* and lungs were harvested at 72-hrs post-infection. Single cells suspensions were stained and analysed by FACS. Neutrophil populations were identified based on their CD11b^+^Ly6G^+^ phenotype. Total cell numbers in the lung were determined based on the number of stained cells. Infection with PA14Δ*hmgA* was marked by a significant increase in the number of neutrophils in the lung compared to mice infected with PA14. The significances of differences between strains were determined by ANOVA * p<0.05 vs. PA14 WT.

We compared the TNF-α response in the mouse lung for AES-1R and AES-1M and separately, PA14/ and PA14-Δ*hmgA* using a TNF-α ELISA. Infection with AES-1M elicited a significantly higher TNF-α response than infection with AES-1R at both 3 and 24 hrs post infection (p<0.001). Even with no detectable lung bacteria at 72 hr, both AES-1R and AES-1M mice still displayed a considerable TNF-α response (ca. 400–500 pg/ml) (Fig 3A). The TNF-α response against PA14 wild type and PA14Δ*hmgA* (Fig 3B) was rapid by three-hrs post-infection, but there was no statistically significant difference in TNF-α response between PA14Δ*hmgA* and PA14 wild type at any time point.

#### 3.3.3 Immunophenotype of PA14Δ*hmgA*

Immune cell counts were conducted on lung and spleen tissue from PA14 and PA14Δ*hmgA*. Single cell suspensions were prepared at 24- and 72-hrs post-infection and analysed by FACS. Cells were stained for the surface markers CD45.2, B220, CD4, CD8, CD11b and Ly6G, to give an overview of the immune cell populations in the lungs of mice infected with 10^6^ CFU PA14 and the respective knockout mutants. At 24 and 48-hrs post-infection, none of the immune cell populations were significantly different between PA14 wild type and PA14Δ*hmgA*. Mice infected with PA14Δ*hmgA* did have a higher total neutrophil count in the lung however this was not statistically significant. By 72-hr post-infection (Fig 3C), mice infected with PA14Δ*hmgA* displayed a significantly higher (p<0.01) lung neutrophil count than PA14 wild type and the uninfected control mice.

There were no statistically significant differences in B cell, CD4 or CD8 T cell populations between PA14Δ*hmgA* and PA14 wild type (data not shown) and despite the observed modulation of some immune cell populations in the lung by PA14Δ*hmgA*, there were no statistically significant changes in population number or frequency observed in the spleen (data not shown).

## Discussion

Previous reports suggest that mutational deactivation of *hmgA* and pyomelanin overexpression is associated with chronic *P*. *aeruginosa* strains infecting CF patients [17], however chronic infection isolates of AES-1 still express *hmgA* (Table 1) and no pyomelanin, indicating putative regulatory control of *hmgA* in chronic *P*. *aeruginosa* AES-1 to suit the host environment rather than adaptive mutation. This downregulation of *hmgA* is not likely a response to the anaerobic/microaerophilic conditions of subsurface growth in CF sputum or sputum-like media, since it also occurs in aerobic growth conditions. On the other hand, AES-1M exhibited greater anaerobic growth compared to AES-1R in ASMDM [10], penetrating into the media with deeper projections by 72 hrs post inoculation, supporting the idea that a consequence of *hmgA* repression may be an increased ability to thrive in a microaerobic/hypoxic environment, and thus persist better [26].

Pyomelanin-containing supernatant is known to protect bacteria from oxidative stress [17], and PA14Δ*hmgA* killed the nematodes at a greatly accelerated rate probably due to this protective effect. The chronic AES-1M isogen showed a non-significant reduction in nematode killing compared to the acute AES-1R despite neither isogen demonstrating accumulation of pyomelanin, indicating that other factors other than *hmgA* and pyomelanin may be involved in protection of this strain Furthermore, the presence of pyomelanin to protect *P*. *aeruginosa* from oxidative stress [27] would be expected to reduce clearance and increase bacterial load in *C*. *elegans*, thus increasing the death rate. In fact, our results show the death rate was reduced in AES-1M assays compared to AES-1R, despite a lack of pyomelanin in both isogens. However as demonstrated in the phenotypic assays, AES-1M underwent a general downregulation of several classic virulence factors compared to AES-1R, while PA14Δ*hmgA* did not downregulate these factors, and this would be expected to contribute to the observed differences in nematode killing.

In contrast, the chronic AES-1M was significantly more persistent in C57Bl/6 mouse lung than its acute counterpart AES-1R at 24 hrs (Fig 2A). In the TNF-α assay, the chronic isogen AES-1M elicited a significantly greater response compared to AES-1M. As TNF-α plays a central role in the initial host response to infection, this higher response to the chronic strain is unexpected. The higher TNF response to 1M suggests that the host is unable to eliminate the infection. This is consistent with the greater persistence shown by AES-1M in the mouse lung. It is also consistent with the amount of inflammatory damage that is seen in the lungs of CF patients due to a prolonged excessive immune response. On the other hand the lack of significant difference in TNF response between PA14 and PA14*hmgA* suggests that absence of this gene alone is not sufficient to induce a detectable difference in host response. PA14*hmgA* induced a significantly stronger neutrophil response compared to wildtype PA14. Importantly, the complemented PA14Δ*hmgA*::*hmgA* demonstrated a phenotype similar to that of the wild type, indicating that the observed changes in PA14Δ*hmgA* are indeed due to mutation of *hmgA*. The finding that wildtype PA14-infected mice displayed greater clinical signs of illness than PA14*hmgA*-infected mice may be a sign that *hmgA* expression elicits a greater clinical manifestation of acute illness.

The novel combination of two mutation methods developed in this study, namely utilizing a combination of the lambda RedS system and a gentamicin cassette flanked by FRT sites, proved a significant improvement on previously established methods [19, 20] as it allowed for the efficient generation of unmarked *P*. *aeruginosa* mutants. Several mutants, including the Δ*hmgA* presented here were successfully generated in a PA14 WT background. Since PA14-like clones have also been isolated from the CF lung, PA14 can be considered to resemble other CF lung-adapted strains in its phenotypic and genotypic characteristics. Additionally, previous studies have used the PA14 strain to establish growth criteria in ASMDM and CF sputum [10, 23, 24].

For future studies, the downregulation but not deactivation of *hmgA* in chronic AES-1 provides a starting point for further investigation, centred on increased hypoxic growth as an consequence of the significant *hmgA* downregulation [9]. AES-1 is transmissible patient to patient [6], and can re-emerge as an acute infection isolate. Therefore by down-regulating *hmgA* rather than deactivating it, AES-1 is in a position to revert to ex vivo aerobic growth when required. As the chronic isogen it also has the ability to better persist in mouse lung but is less lethal to the host (*C*. *elegans*).

In conclusion, the results point to deactivation of *hmgA* resulting in a phenotype characteristic of an organism that has successfully adapted to the niche environment in the CF lung, but also that *hmgA* downregulation without pyomelanin production does not hamper adaptation and persistence. Within the CF lung environment, these results suggest that downregulation of *hmgA* may be more likely to result in a long-term infection, while deactivation appears coupled with an ongoing excessive neutrophil response, two conditions which are conducive to significant tissue damage within the lung and poor patient outcomes [9, 28–30]. However, it is unknown if deactivation (as opposed to downregulation) affects a particular strains’ ability to survive ex vivo and to reinfect other CF patients. Other genes would also be involved in the ability to re-infect a CF patient, and their regulation at acute and chronic infection stages would need to be investigated. Ideally it would be useful to be able to mutate *hmgA* in AES-1 however this clinical strain has proved extremely intractable. It is highly likely that deletion or downregulation of *hmgA* may also have other currently unknown effects, and as such it is an attractive gene target for further studies characterising the role it may play in inducing the inflammatory response and the downstream regulatory effects identified here.

This study has identified and outlined a putative role for *hmgA* downregulation in chronic *P*. *aeruginosa* AES-1 from the cystic fibrosis lung, namely, by not being deactivated *hmgA* can be re-expressed during acute re-infection of another CF patient by this transmissible strain. The results of this study form the basis for further investigations to identify and characterise the genes that are important in the pathogenesis and persistence of *P*. *aeruginosa* AES-1 in the cystic fibrosis lung.
