# Correction: Homogentisate 1-2-Dioxygenase Downregulation in the Chronic Persistence of *Pseudomonas aeruginosa* Australian Epidemic Strain-1 in the CF Lung

**DOI:** 10.1371/journal.pone.0138633

**Published:** 2015-09-14

**Authors:** The PLOS ONE Staff

The image for Fig 3 is incorrect. The publisher apologizes for the error. Please see the corrected Fig 3 here.

**Fig 3:**
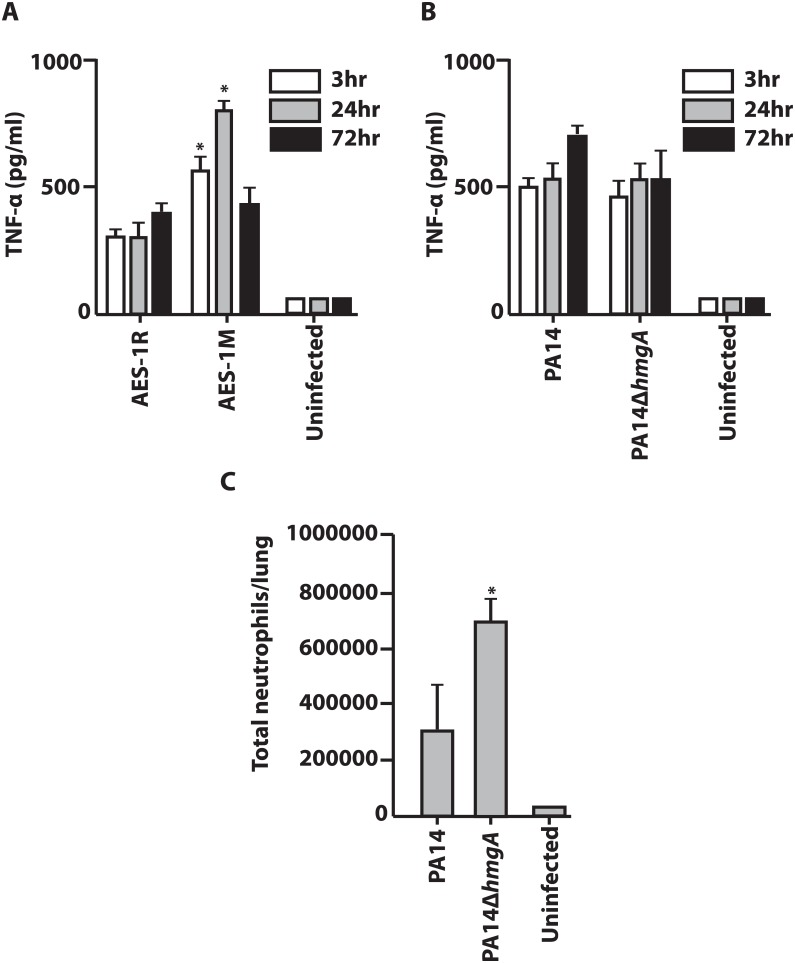
Mouse TNF-α response to lung infection with AES-1R, AES-1M, PA14 and PA14Δ*hmgA*, and neutrophil populations in PA14 and PA14Δ*hmgA*-infected mice. The TNF-α profile of *P*. *aeruginosa*-infected mice was determined in lung homogenates at 3-, 24- and 72-hrs post-infection by ELISA. A. AES-1M elicited a rapid and significantly greater TNF-α response compared to AES-1R at 3 and 24-hr, and both responses were still at >400pg/ml at 72hr despite the absence of bacteria in lungs. B. Both PA14 and PA14*hmgA* elicited a rapid TNF-α response compared to control uninfected mice by three hrs post-infection however there was no significant difference between them. The significances of differences between strains were determined by ANOVA * p<0.0001 vs. AES-1R. C. Mice were infected with 10^6^ CFU of either wildtype PA14 or PA14Δ*hmgA* and lungs were harvested at 72-hrs post-infection. Single cells suspensions were stained and analysed by FACS. Neutrophil populations were identified based on their CD11b^+^Ly6G^+^phenotype. Total cell numbers in the lung were determined based on the number of stained cells. Infection with PA14Δ*hmgA* was marked by a significant increase in the number of neutrophils in the lung compared to mice infected with PA14. The significances of differences between strains were determined by ANOVA * p<0.05 vs. PA14 WT.

## References

[1] Harmer CJ, Wynn M, Pinto R, Cordwell S, Rose BR, Harbour C, et al (2015) Homogentisate 1-2-Dioxygenase Downregulation in the Chronic Persistence of *Pseudomonas aeruginosa* Australian Epidemic Strain-1 in the CF Lung. PLoS ONE 10(8): e0134229 doi: 10.1371/journal.pone.0134229 26252386 10.1371/journal.pone.0134229 PMC4529145

